# The Modern Treatment of Syphilitic Diseases

**Published:** 1854-09

**Authors:** 


ART. VI.—*The Modern Treatment of Syphilitic Diseases, both Primary and Secondary;* comprising the Treatment of Constitutional and Confirmed Syphilis by a safe and successful method; with numerous cases, formulæ, and Clinical observations. By LANGSTON PARKER, Surgeon to the Queen's Hospital, Birmingham. From the third, and entirely re-written London edition. Philadelphia: Blanchard & Lea. 1854.

---

The author of this treatise remarks, in his preface, that he "has now devoted nearly twenty years to the Therapeutics of Syphilis, more especially in its secondary and constitutional forms." In this time, without taking into consideration the extended field afforded him by a large hospital, he has personally treated more than eight thousand cases, and the results of his large experience are recorded in this work. Such a work could hardly fail to be valuable in an uncommon degree to the practitioner.

One would conclude from the number of treatises lately published in this country on the class of diseases to which this refers, that the subject was pretty thoroughly exhausted; but we believe it will turn out that the uncertainty which hangs over some of the more important topics pertaining to syphilis is in direct proportion to the multiplicity of the publications on the subject. In truth, we are not acquainted with a medical question concerning which a greater contrariety of opinion prevail than some questions relating to this disease. The most important question of all—the best mode of treating it—is by no means settled. Is mercury necessary or advisable in the treatment of these affections, is a question to which a different answer would be given by different practitioners. And equally diverse are the views of surgeons in regard to the nature of the disorder. HUNTER, as is well known, perpetuated the doctrine down to our own times, that gonorrhea and syhpilis are forms of the same disease. Being held as the same, the treatment was for a long time the same in both, and so late as the period when Sir ASTLEY COOPER was appointed to Guy's Hospital, patients suffering from gonorrhea, we are told by him, were compelled to rub in so many drachms of mercurial ointment till a profuse salivation was induced. The views of HUNTER were opposed in his time by BENJAMIN BELL, of Edinburgh, but the popularity of the great London Surgeon bore down all opposition. Sir CHARLES BELL, the late professor of surgery in the University of Edinburgh held that a father would be unnatural who would submit a son with syphilis to any treatment except a mercurial one. But the present professors of that University entirely repudiate mercury in the treatment of all forms of syphilis, both primary and secondary.

From this it will be perceived that there are important points to be settled in respect to this disorder, and we regard the contribution of Mr. PARKER to the literature on the subject as timely and important. Both extremes of opinion held by the two schools, we have no doubt, are equally pernicious. One object of this work is to prove this, and we think the author makes it clear that entire abstinence from mercury in syphilis is as irrational as its indiscriminate use in all the varieties and stages of that affection. He remarks that "syphilis is modified and complicated in a variety of ways by the age, habits, and constitution of the patient; and that treatment can only be rational, successful, and safe, which is eclectic, and founded upon the actual nature and peculiarities of each individual case."

Of the two modes of treating syphilis—the simple, and the mercurial—Mr. PARKER thus briefly speaks:—

"The non-mercurial, simple, or physiological treatment of syphilis, then, consists in the employment of the means already passed in review, both local and constitutional, without having recourse to mercury as a specific therapeutic agent in their cure, and this may be adopted both in the primary and secondary forms of disease. It will be found, however, that the primary are very much more easily cured than the secondary upon such a plan. It cannot be concealed, that the non-mercurial treatment does not always succeed in the cure of primary syphilis; and that, in a great number of cases, the cures are more apparent than real, the sores breaking out again when the patients return to their customary diet and occupations. Matters go on very well whilst a patient is limited to a rigid diet, and confined to bed, and watched in the wards of a hospital, but in private practice this cannot be done; and hence it has been found by military surgeons, that whilst they could cure the privates, they could not cure the officers on the non-mercurial plan. In the French memoirs of military surgery, the medical officers of the military hospital of Toulon state that, although the non-mercurial plan is useful in allaying the irritation or inflammatory symptoms which accompany primary venereal sores, yet they were compelled to resort to mercury to obtain radical cures. Fifty-two surgeons met at Nantes, in July, 1835, to discuss this question: they had five discussions; two only, one of whom was M. Devergie, declared themselves in favor of the physiological, or non-mercurial treatment of syphilis.

"As a general rule or principle, I never employ mercury except as an aperient, in the ordinary forms or earlier stages of primary venereal sores, except such sores have been tested by inoculation, and yielded a characteristic pustule. The immediate local or specific effect of the syphilitic virus produces a degree of irritation or inflammation on the parts to which it is applied, during the continuance of which mercury is, to say the least, injurious, except as an aperient; and it is not till rest, low diet, mild opiate or astringent washes, and other remedies just noticed, have failed in producing a cure, that mercury is to be thought of as a specifie agent. When, however, all these have failed, and the case has assumed a perfectly chronic character, mercury may be used with every prospect of a beneficial result, and this is certainly the result of modern experience on this subject."

In regard to the best method of introducing mercury into the system, he remarks:—

"The treatment by friction is much more certain than by the internal administration of mercury. This method consists in rubbing in before the fire each night, till the proper effect is produced. from a scruple to a drachm of the stronger mercurial ointment. These frictions may be made on the inside of the thighs, in the popliteal space, on the soles of the feet, or in the axillæ. Frictions in the axillæ are of service in obstinate ulcerations of the throat. I very frequently employ them with complete success in this situation. Cullerier records the histories of two cases cured by mercurial friction in the axillæ, which had resisted its employment in other parts. Sir B. Brodie, (Lectures on Pathology and Surgery, p. 242,) prefers this method, which was that of the late Mr. Pearson of the Lock Hospital, to all others; he believes that surgeons have gone back in their treatment of syphilis. Mr. Hunt (On Syphilitic Eruptions with especial reference to the use of Mercury) is of the same opinion. In reference to the treatment of infantile syphilis, it is clear that frictions are much more safe and efficacious than treatment by the mouth. "Very few of those children ultimately recover to whom mercury has been given internally; but I have not seen a single case in which the other method of treatment has failed." The frictions must be continued every night, or every other night till the gums swell, and the secretions of saliva is slightly increased; the intervals between the frictions must then be lenthened, but the effect kept kept up till sometime after the sore has healed, and its specific induration gone. We cannot, with Chelius and others, limit the number of frictions to nine, or twelve, or any specific number, they must be continued till the effects above mentioned are produced. That mercurial treatment by friction or by the mouth should succeed, it is absolutely essential that the patient should be confined to a warm room, and if possible to bed; exposure during a mercurial course sometimes entirely destroys the effect of the remedy. It is owing to an observance of these rules that patients in the hospital get cured, who are made to observe them, whilst private patients who neglect them or cannot observe them, whilst private patients who neglect them or cannot observe them, are not so fortunate. The diet during this treatment should be light, nutritious, and unstimulating; the older and more weakly the patient the better the diet; should he be young and plethoric, he should combine the hunger cure with the frictions. The treatment by friction is milder, safer, and more certain than that by the internal administration of mercury; but it is liable occasionally, though not so frequently, to produce the evils already alluded to when speaking of the use of that class of remedies. In certain cases, frictions of different preparations of mercury have been made on the gums and upon the tongue. I have tried them, but at best with little success."

He prefers fumigation in other forms:

"In a great number of primary diseases I prefer the treatment by fumigation, combined with the internal administration of a twentieth of a grain dose of the bichloride or biniodide, and a milk diet. In indurated chancre and primary phagedenic sores, the treatment does not fail, neither is it attended with accidents, such as diarrhœa, or salivation, once in a hundred times; it is as certain and as little hurtful as any treatment can possibly be.

"Between the years 1846 and 1850 I personally treated fifty-eight cases of indurated chancre in this way; none of the patients were confined by the treatment, though I admit this would have been better could it have been accomplished, but in a great majority of syphilitic cases this is impossible; it is therefore our duty to frame some treatment that will be efficacious without such an important auxiliary. Diet can be observed, but rest in bed or confinement to a warm room cannot with a great mass of private patients. One case only out of the fifty-eight, up to the present period, has been followed by secondary symptoms, and that in the form of slight lepra with superficial ulceration of the throat, which occurred in three months after the primary disease. In five of these cases the sores were situated at the orifice of the urethra, and in one accompanied also by phymosis, the glans and prepuce were as hrrd as a scirrhous mamma; nevertheless the cure was perfect and the health unimpaired, although the patient was nearly fifty years of age.

"In primary ulcerative (not sloughing) phagedena, I have not as yet seen one single instance of failure. Salivation rarely accompanies treatment by moist fumigation, and this is prevented by the profuse sweating which the process occasions. If patients use an ordinary vapor bath whilst taking mercury internally, or employing it by friction, salivation very rarely takes place."

Sound practical sense characterizes every page of this work, and we recommend it with great confidence as a safe guide in the treatment of syphilis. It is full enough in its details for all the purposes of the practitioner, without being unnecessarily encumbered with cases, or discussions on scientific points.

